# Sea food by-products valorization for biomedical applications: evaluation of their wound regeneration capabilities in an *Ex vivo* skin model

**DOI:** 10.3389/fvets.2024.1491385

**Published:** 2024-11-18

**Authors:** Giulia Zivelonghi, Luca Melotti, Anna Carolo, Andrea Venerando, Margherita Roncoroni, Giordana Martinelli, Lisa Maccatrozzo, Stefania Marzorati, Michela Sugni, Marco Patruno

**Affiliations:** ^1^Department of Comparative Biomedicine and Food Science, University of Padua, Padua, Italy; ^2^Department of Agricultural, Food, Environmental and Animal Sciences, University of Udine, Udine, Italy; ^3^Department of Environmental Science and Policy, University of Milan, Milan, Italy

**Keywords:** antioxidants, circular economy, collagen-based wound dressing, *ex vivo* organ culture, innovative therapies, regenerative medicine, skin, wound healing

## Abstract

**Introduction:**

The skin is often exposed to harmful stimuli that might compromise its integrity and functionality. After an injury, the skin has a limited capability to restore its complex structure, and in the case of severe skin damage, surgical operations and rapid application of wound dressings are often required to promote optimal wound healing. Nowadays, collagen-based biomaterials are widely used in combination with bioactive molecules able to prevent excessive inflammation and possible infections. In line with a circular economy and blue biotechnology approach, it was recently demonstrated that both collagen and bioactive molecules (i.e., antioxidant compounds) can be sustainably obtained from sea food by-products and effectively used for biomaterial development. Herein, we describe and compare the application of two marine collagen-based wound dressings (CBWDs), produced with materials obtained from sea urchin food waste, for the treatment of skin lesions in a wound healing organ culture (WHOC) model.

**Methods:**

The *ex vivo* WHOC model was set up starting from rat skin explants and the induced lesions were assigned into three different groups: control (CTRL) group, not treated, marine collagen wound dressing (MCWD) group, and antioxidants-enriched marine collagen wound dressing (A-MCWD) group. After 5 and 10 days, specimens were examined for organ maintenance and assessed for the healing process.

**Results:**

Immunohistochemical results showed that both CBWDs were similarly successful in prolonging skin repair, preserving the epidermal barrier up to 5 days under static culture conditions. Histological and gene expression analysis highlighted that the A-MCWD might support and accelerate skin wound healing by exerting antioxidant activity and counteracting inflammation.

**Discussion:**

Overall, these findings underline the potential of sea urchin food waste as a novel resource for the development of functional medical devices for the treatment of skin wounds.

## Graphical abstract

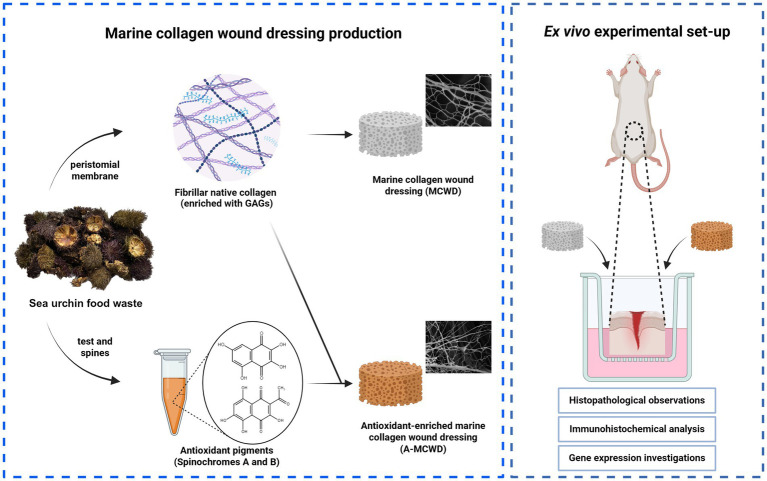

## 1 Introduction

Skin represents the largest organ of the body in vertebrates (1). Its highly complex structure enables numerous processes involved in maintaining body homeostasis, such as the defense against external insults as well as prevention of infections (2) and fluid losses; also, the peculiar microarchitecture of the skin confers it an important role in sensory detection and self-healing (3–5). Skin wound healing is a complex and dynamic physiological process that aims at restoring tissue integrity and functionality following injury (6). It consists of four interrelated and overlapping phases (hemostasis, inflammation, proliferation, and tissue remodeling) that are governed by cell–cell, extracellular matrix (ECM) and soluble factors interactions (7–9). Most of the time, skin wounds are able to heal on their own. However, severe injuries of the skin often require surgery and moist dressing application to reach proper healing (10, 11). Given this, the management of major skin traumas such as deep partial-thickness (DPT) or full-thickness (FT) skin injuries represents one of the most challenging issues in clinical practice (12, 13). Currently, the gold standard for treating extensive cutaneous defects is autologous skin grafting: an auto transplantation procedure that has evolved over the years into new therapeutic modalities using tissue grafts or flaps (14) (e.g., the setup of dotted skin grafts for the treatment of diabetic foot ulcers (15, 16)) but is still limited by donor site issues. For example, the lack of sufficient healthy tissue (e.g., in the case of extensive injuries (17) or due to genetic-driven conditions (18)), hinders the skin autografting procedure. Furthermore, it should be considered that when the treatment is feasible, the donor site becomes a wound itself. Finally, skin autografting often results in non-functional (scar) tissue since areas with extensive dermal defects fail to support the graft (19–22). In response to such limitations, over the past decades regenerative medicine and tissue engineering have been investigating alternative methods to the conventional wound management in clinical practice, mainly focusing on the development of skin biomaterials, such as wound dressings or skin substitutes, for the treatment of severe skin loss. Indeed, wound dressings cover the injured skin and maintain a moist environment with the appropriate conditions for healing, providing physical and microbiological protection, preventing excessive fluid loss and relieving pain. Notably, most recent wound dressings may also contain natural extracts with anti-inflammatory, epithelializing, antioxidant, or antimicrobial properties (23–26). Among these, collagen-based wound dressings (CBWDs) have been used extensively to provide adequate wound coverage and have become some of the most widely used wound dressings available on the market, such as Apligraf^®^ (27), OrCel^®^ (28) or Integra^®^ Matrix wound dressing (29, 30). More recently, following the principle of circular economy and adopting a blue biotechnology approach, research has moved toward the production of marine-derived collagen biomaterials obtained from sea food wastes as an alternative and sustainable wound care solution to commercial biomedical devices of mammalian origin, thus contributing to progress in the field of circular economy and regenerative medicine (31–34). Compared to the latter, the benefits of sea-derived collagen include no risk of zoonotic diseases and pathogens for patients, improved chemical and physical durability, and large availability (35). Among all marine invertebrates, sea urchins are a promising source of native collagen, since a considerable percentage of the mass of these echinoderms becomes waste in the food industry. Considering this, some researchers have attempted to recycle the by-products of the mediterranean purple sea urchin (*Paracentrotus lividus*) by recovering the non-edible parts to obtain high valuable molecules of biomedical interest (36–39). Ultimately, GAG-decorated fibrillar collagen extracted from the peristomial membrane was used to produce innovative and eco-friendly three-dimensional CBWDs. This marine collagen wound dressing (MCWD) was then successfully tested *in vivo* in rat and sheep models (40, 41). In addition, antioxidant pigments, namely polyhydroxynaphtoquinones (PHNQs) (42), purified from the test and spines of the same sea urchin by-products, were tested *in vitro* for their antioxidant activity (43). Due to their promising biological properties, PHNQs were also incorporated into MCWD to obtain the antioxidant-enriched marine collagen wound dressing (A-MCWD). Herein, with the aim of limiting the use of animals, the *ex vivo* organ culture (EVOC) was used as a valuable preclinical model to evaluate the effects of novel therapies for skin wound repair. EVOC systems are already used to investigate the properties of topical pharmaceutical excipients through skin. However, studies using skin EVOC were focused on superficial injuries of human skin (44–48). Nevertheless, it has been reported that skin EVOC can be cultured for more than 9 days without any undesirable physiological effects (49). Considering the absence of a standardized protocol for an *ex vivo* animal skin model, in this work we describe, for the first time, the application and comparison of the MCWD and A-MCWD CBWDs for the treatment of deep skin injuries (i.e., partial thickness injuries) establishing a rodent wound healing organ culture (WHOC) model.

## 2 Materials and methods

### 2.1 Collagen-based wound dressings (CBWDs) production

#### 2.1.1 Sea urchin waste

Both sea urchin collagen and polyhydroxynaphtoquinones (PHNQs) were extracted from *Paracentrotus lividus* waste, kindly donated by restaurants near the University of Milan after gonads removal (the only edible part of the animal). The waste was kept frozen at −20°C until use. Collagen was specifically extracted from the peristomial membranes (i.e., the soft tissue around the animal’s mouth), while PHNQs were extracted from the remaining part of the waste (namely from tests and spines). These ones were lyophilized for 24 h to remove residual water (5Pascal S.r.l., Milan, Italy). The dried material was powdered and stored at room temperature (RT) until the extraction of PHNQs.

#### 2.1.2 Collagen extraction

Collagen was extracted according to a previously published method (34, 35). Briefly, the peristomial membranes were rinsed with artificial seawater, weighed and left overnight at 23°C in a hypotonic solution (10 mM Tris–HCl, 0.1% EDTA, pH 8.0) under stirring conditions. After several washes with phosphate-buffered saline (PBS), a decellularizing solution (10 mM Tris–HCl, 0.1% SDS, pH 8.0) was added and the samples were left overnight at 23°C with stirring. After removal of the SDS with PBS, the samples were treated with a disaggregating solution (0.5 M NaCl, 0.1 M Tris–HCl pH 8.0, 0.1 M *β*-mercaptoethanol, 0.05 M EDTA) for 5 days at 23°C under stirring conditions. The collagen suspension was filtered through a steel mesh filter (0.2 mm), dialyzed against 0.5 M EDTA for 4 h and then dialyzed against distilled water overnight to completely remove residual *β*-mercaptoethanol. The purified collagen suspension was stored at −80°C.

#### 2.1.3 Polyhydroxynaphtoquinones extraction

The extraction of PHNQs from lyophilized sea urchin powder was optimized in our laboratories (38) as follows: for 50 g of lyophilized sea urchin powder, 175 mL of formic acid solution (6 M) was added dropwise, and the mixture was stirred for 2 h at room temperature. The resulting suspension was centrifuged at 4000 *g* for 5 min (Eppendorf, Centrifuge 5804 R) and the supernatant was filtered under vacuum. Liquid–liquid counter-extraction was performed three times with 150 mL aliquots of fresh ethyl acetate. To remove inorganics salts, the resulting organic phase was counter-extracted with fresh milli-Q water until the conductivity and pH of the aqueous phase were similar to those of milli-Q water. Anhydrous sodium sulfate was added to remove residual water and finally the extract was dried using a rotary evaporator (37°C) and a mechanical vacuum pump.

### 2.2 Preparation of wound dressings (MCWD and A-MCWD CBWDs)

The collagen suspension was centrifuged at 1700 *g* for 5 min and resuspended in a 6% (v/v) EtOH/water solution to give a 6 mg/mL suspension. A PHNQ-collagen suspension was prepared by adding PHNQ at 1% (w/w) relative to collagen. To prepare the MCWD and A-MCWD, 1 mL of collagen suspension and PHNQ-collagen suspension, respectively, was added to a silicone rubber mold (1.5 cm diameter, 0.5 cm height), frozen at −80°C for 3 h and lyophilized.

### 2.3 Animal model and ethical statement

Male Sprague Dawley rats (*Rattus norvegicus*) were allocated in an animal facility (Department of Neurosciences-DNS, Human Anatomy Institute, University of Padua, Padua, Italy) into different cages for acclimating themselves for 2 weeks. During this period, the animals had free access to water and feed, and they were periodically physically examined to assess their health status. The experiment protocol was approved by the Italian Ministry of Health (no 57/2022-PR) in accordance with the Body for the Protection of Animals (OPBA) of the University of Padua. For the *ex vivo* experiment, healthy rats, sacrificed for other experimental reasons, were euthanized with a gas overdose (carbon dioxide) in the euthanasia induction chamber. To prevent eventual wound infection, favor skin regeneration, and to facilitate the successive phases of wound healing analysis, the excess of dorsal hair was removed with an electric shaver. Subsequently, the skin of the entire dorsum was excised surgically in aseptic conditions and immersed in PBS (Sigma Merck, Darmstadt, Germany) solution containing 10% Penicillin and Streptomycin (P/S; Sigma Merck, Darmstadt, Germany) for transport. The obtained skin explants, measuring approximately 9 cm × 9 cm, were stored at +4°C for a maximum of 4 h.

### 2.4 Wound healing organ culture (WHOC) model

The skin explants were used on the day of tissue collection. All procedures were performed in sterile conditions. After removing the excess of adipose tissue, skin samples were washed twice in PBS 1X solution to remove residual blood. A total of 56 skin discs were created using a 6 mm biopsy punch. On each of them, a deep partial-thickness (DPT) incisional wound was performed mechanically by gently applying a 2 mm biopsy punch. During these procedures, the skin samples were maintained in PBS 1X solution until culturing. The resulting skin discs were randomly assigned into three different groups: 16 in the control group (CTRL), which was untreated; 16 in the marine collagen wound dressing (MCWD) treated group; 16 in the antioxidant-enriched marine collagen wound dressing (A-MCWD) treated group. Two timepoints, at day 5 and day 10, were defined for each group for tissue sampling. Timepoint at day 0 was also established for the unwounded group (*n* = 8). Prior to setting up the WHOC model, the MCWD and A-MCWD CBWDs were sterilized for 45 min per side under Ultraviolet (UV) radiation in a biosafety cabinet in sterile conditions. Next, the dressings were shaped with the use of a 2 mm biopsy punch, and the obtained biomaterials were used to cover the skin lesions. Skin discs were distributed into cell culture inserts (0,4 μm cellstar ThinCert^™^, Greiner Bio-One S.r.l., Milan, Italy) into two 24-well plates and cultured in Dulbecco’s Modified Eagle Medium (DMEM; Sigma Merck, Darmstadt, Germany) supplemented with 10% Fetal Bovine Serum (FBS; Sigma Merck, Darmstadt, Germany) and 1% P/S, and maintained in an incubator at +37°C with 5% CO_2_. The medium was changed every 2 days. At the defined timepoints (5 and 10 days), specimens were properly collected and preserved in 10% neutral-buffered formalin (Kaltek S.r.l, Padova, Italy) at room temperature (RT) for histopathological and immunohistochemical analysis, or in RNA*later*^®^ (Thermo Fisher Scientific, Waltham, MA, USA) following manufacturer’s instructions for gene expression analysis by Real Time PCR (RT-PCR), respectively. Samples for RT-PCR were stored at −80°C until analyzed. At 0, 5 and 10 days, the skin discs were placed on a sterile surgical towel for macroscopic examination.

### 2.5 Histopathology and image analysis

At all timepoints, samples were fixed in 10% neutral-buffered formalin at RT for at least 16 h. Afterwards, they were dehydrated by using a gradual dilution of ethanol and embedded in paraffin following standard procedures. Afterwards, the wax-coated samples were cut with a Leica—RM2035 (Leica Microsystems, Wetzlar, Germany) microtome into 5 μm thick slices. Sections were stained with Harris’ hematoxylin and eosin (H&E) and Masson-Goldner’s Trichrome (MT) following standard protocols. Digital images were obtained using a ZEISS Axioscan 7 whole slide scanner (Carl Zeiss Microscopy GmbH, Jena, Germany). H&E-stained sections were measured for total epidermal thickness. From each specimen, eight fields along the epidermis were acquired at 200× magnification using the free software ZEISS ZEN 3. The RGB images were subjected to morphometric analysis using ImageJ software. In total, 40 measurements for each sample were taken to determine the thickness of the epidermis.

Collagen positive area was quantified in the MT-stained sections using the ImageJ software. For image analysis, six regions of interest (ROI) were randomly selected in each sample. Morphometric analyses were performed using ImageJ software to measure collagen fiber density following a modified protocol proposed by Hong and colleagues (50). Briefly, color thresholding tool was used to identify the light green pixels (i.e., collagen) keeping the same settings for each image. The amount of collagen fibers was calculated as a percentage of the total area.

### 2.6 Immunohistochemistry and image analysis

Skin samples were processed as described for histopathology. For immunohistochemical staining, 4 μm thick sections were labeled with anti-Cytokeratin 10 (Ab76318; Abcam, Cambridge, UK), anti-Cytokeratin 14 (Ab181595; Abcam, Cambridge, UK) and anti-Proliferation marker protein Ki-67 (Ab16667; Abcam, Cambridge, UK) antibodies (Table 1). If required, antigen retrieval was performed by heat induced epitope retrieval (HIER) in either Tris-EDTA Buffer, pH 9.0 (10 mM Tris Base, 1 mM EDTA) or Sodium Citrate Buffer, pH 6.0 (10 mM Sodium citrate, 0.05% Tween 20). In order to avoid false positive signals, endogenous peroxidase activity was blocked by exposing tissue sections to 0.3% hydrogen peroxide solution at RT for 20 min. Nonspecific binding sites were blocked by incubating sample sections to 2.5% normal horse serum (ImmPRESS^®^ HRP Universal Polymer Kit, Peroxidase, Horse Anti-Mouse/Rabbit IgG; Vector Laboratories, California, United States) for 20 min at RT. Sections were then incubated with the primary antibody diluted in PBS 1X solution at 4°C, different time of incubation for each antibody were set (see Table 1). Afterwards, samples were washed three times (5 min each) in PBS and incubated with a secondary antibody-HRP conjugated (ImmPRESS^®^ HRP Universal Polymer Kit, Peroxidase, Horse Anti-Mouse/Rabbit IgG; Vector Laboratories, California, United States) at RT for 30 min. Finally, samples were washed twice in PBS (5 min each) and exposed to DAB solution (DAB Substrate Kit, peroxidase with nickel; Vector Laboratories, California, United States). All skin sections were counterstained with Mayer’s hematoxylin solution (PanReac AppliChem ITW Reagents, Monza, Italy). The washing steps were performed with PBS 1X, or a solution of Tris buffered saline 1X and 0.05% Tween^®^ 20 detergent (P1379, TWEEN^®^ 20 viscous liquid; Sigma Merck, Darmstadt, Germany) (TBST 1X) (Table 1). Positive and negative controls were always performed in parallel with experimental sections to assess the specificity of immunostaining reaction. Digital images of stained slides were obtained using a whole slide scanner (ZEISS Axioscan 7) fitted with a Plan-Apochromat 40x/0.95 Korr M27 objective lens (Carl Zeiss Microscopy GmbH).

**Table 1:** Primary antibodies used for immunohistochemical staining.

Primary antibody	Code	Antigen retrieval	Dilution	Incubation time	Washing step
Cytokeratin 10 (K10)	Ab76318	HIER Tris-EDTA Buffer pH9	1:3500	2 h	PBS 1X
Cytokeratin 14 (K14)	Ab181595	Not required	1:2000	2 h	PBS 1X
Proliferation marker protein Ki-67 (Ki67)	Ab16667	HIER Sodium Citrate Buffer pH6	1:100	overnight	TBST 1X

The DAB-positive areas of the immunohistochemical (IHC-P) Cytokeratin 10 (K10)- and Cytokeratin 14 (K14)-stained sections were quantified using the ImageJ software according to a modified protocol (51). Eight fields along the epidermis were acquired from each specimen at magnification 400×. Quantitative analysis of the DAB-positive areas were performed using Color Deconvolution2 tool on ImageJ. The DAB-positive area was calculated as percentage of total area. For the anti-Ki67-stained sections, a semi-quantitative analysis was performed as described in (52, 53). Briefly, Ki67-positive cells were counted, and the overall staining intensity was determined. A histological score was given using four categories of evaluation: negative (0), weak (+), moderate (++), and strong (+++).

### 2.7 Gene expression analysis

Skin samples stored in RNA*later*^®^ were processed for total RNA extraction. After removing the RNA*later*^®^ solution, samples were immersed in TRIzol reagent (Life Technologies, Carlsbad, CA, USA) and homogenized using silica beads in a benchtop tissue lyser (TissueLyser II, Qiagen Inc., Hilden, Germany). RNA was extracted following the manufacturer’s protocol. Then, the RNA extracted was assessed for its quality and quantified using a Nanodrop spectrophotometer (Thermo Fisher Scientific, Waltham, MA, USA). RNA samples were stored at −80°C until use. A total amount of 500 ng of RNA was retrotranscribed with Superscript^™^ II Reverse Transcriptase (Invitrogen, Carlsbad, CA, USA) to obtain complementary DNA (cDNA). Thus, the obtained cDNA was used as template for the real time polymerase chain reaction (RT-PCR) gene expression analysis using the ABI 7500 Real-Time PCR system (Applied Biosystems, Foster City, CA, USA). 96-well PCR plates were used, and samples were run in duplicate. In this study, the relative expression of genes involved in the wound healing process was evaluated using specific pairs of primers, which were designed using the Primer Express 3.0 software (Applied Biosystems, Foster City, CA, USA). The sequence of primers was designed based on the rat annotated genome sequence on the GenBank database (rat genome assembly: GCA_015227675.2) (Supplementary Table S1). The efficiency of the designed primers was assessed by using the standard curve method. All pairs of primers presented an acceptable slope (between −3.8 and −3.3) with a corresponding efficiency of 90–100%. To calculate the efficiency, the ABI 7500 System SDS Software v1.5.1. (Applied Biosystems, Foster City, CA, USA) was used. The relative expression of genes involved in the skin wound-healing process was evaluated: COL1A1 (Collagen Type I Alpha 1 Chain), COL3A1 (Collagen Type III Alpha 1 Chain), GPx1 (Glutathione peroxidase 1), IL-6 (Interleukin 6), KGF or FGF7 (Keratinocyte Growth Factor, or Fibroblast Growth Factor 7), KRT16 (Keratin 16), MMP-9 (Matrix Metallopeptidase 9), NOX1 (NADPH Oxidase 1), PDGFB (Platelet Derived Growth Factor Subunit B), SOD2 (Superoxide Dismutase 2), TNF (Tumor Necrosis Factor), TIMP-2 (Tissue Inhibitor of Metalloproteinase 2), VEGF (Vascular Endothelial Growth Factor A). The TATA-Box Binding Protein (TBP) and Ribosomal Protein Lateral Stalk Subunit P0 (RPLP0) were used as reference genes to normalize the obtained data. All experiments were run in duplicate to study the relative gene expression of each gene of interest. A melting curve analysis (dissociation curve) was performed as well to detect the non-specific amplification.

The relative expression was calculated by using the 2^−∆∆Ct^ method to normalize the cDNA level of expression of the gene of interest to the reference genes. The uninjured skin (day 0) was used as the calibrator sample.

### 2.8 Statistical analysis

All data are expressed as mean ± standard error of the mean (SEM). Before performing all statistical analysis, data were assessed for their Gaussian distribution by normal distribution by applying the Shapiro–Wilk normality test. Statistical differences were evaluated using the two-way ANOVA test with the Tukey’s “post-hoc” test to detect statistical differences (*p* ≤ 0.05) in all groups. Data were analyzed using GraphPad Prism 9.0 software (San Diego, CA, USA). Comparison among groups regarding the epidermal thickness was performed using the Kruskal-Wallis nonparametric test by time using the XLSTAT software package (Data Analysis and Statistical Solution for Microsoft Excel, Addinsoft, Paris, France). Differences showing a *p*-value lower or equal to 0.05 were considered statistically significant.

## 3 Results

### 3.1 Wound dressing preparation and *ex vivo* model set up

**Figure 1:**
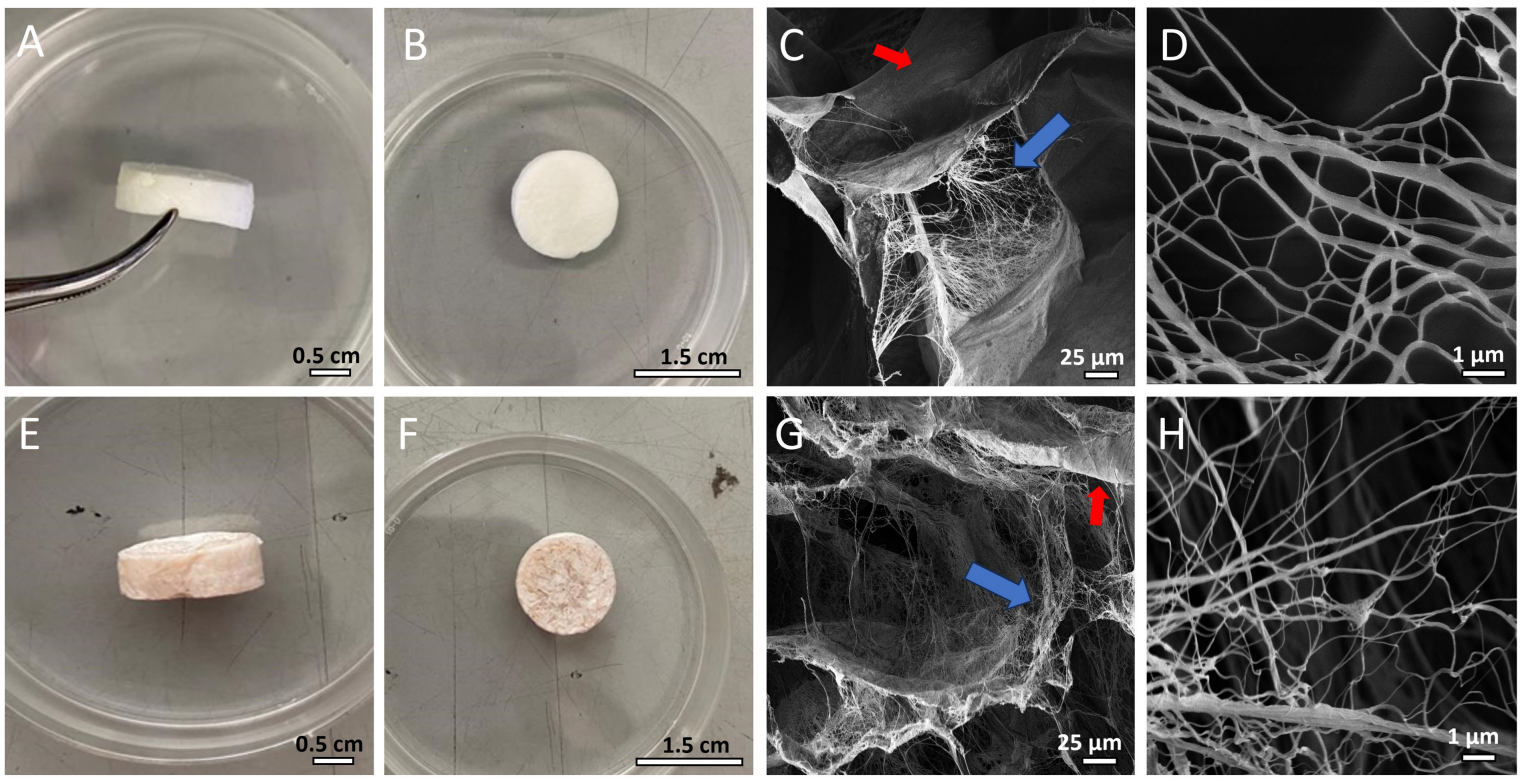
**(A,B)** MCWD biomaterial. **(C)** SEM image of MCWD (blue arrow: fibril network; red arrow: laminar structure). **(D)** SEM image of MCWD collagen fibrils. **(E,F)** A-MCWD biomaterial. **(G)** SEM image of A-MCWD (blue arrow: fibril network; red arrow: laminar structure). **(H)** SEM image of A-MCWD collagen fibrils. Other ultrastructural details of the scaffolds are also shown in SEM images in the study by Martinelli et al. (2024, submitted, under review).

After production, both CBWDs appeared as 3D-sponge-like porous cylinders (Figure 1). CBWDs containing PHNQs showed a more reddish/brownish color, due to the presence of pigments (i.e., PHNQs) (Figures 1A,B,E,F). The ultrastructure of both biomaterials was evaluated by scanning electron microscopy (SEM) to investigate in detail the fibrillar network. SEM analyses showed that composites (A-MCWD) (Figures 1G,H) are ultrastructurally similar to the pure collagen-based counterpart (MCWD) (Figures 1C,D), displaying a homogeneous porous/fibrillar structure without visible aggregates. In both biomaterials the fibril network is intermixed with the laminar structure. More detailed information on the physicochemical properties of both biomaterials (ultrastructure, swelling and degradation properties) have already been discussed in Martinelli et al. (2024, submitted, under review).

In addition, the swelling properties and degradation kinetics of A-MCWD and MCWD results are summarized in Supplementary Tables S2, S3.

**Figure 2:**
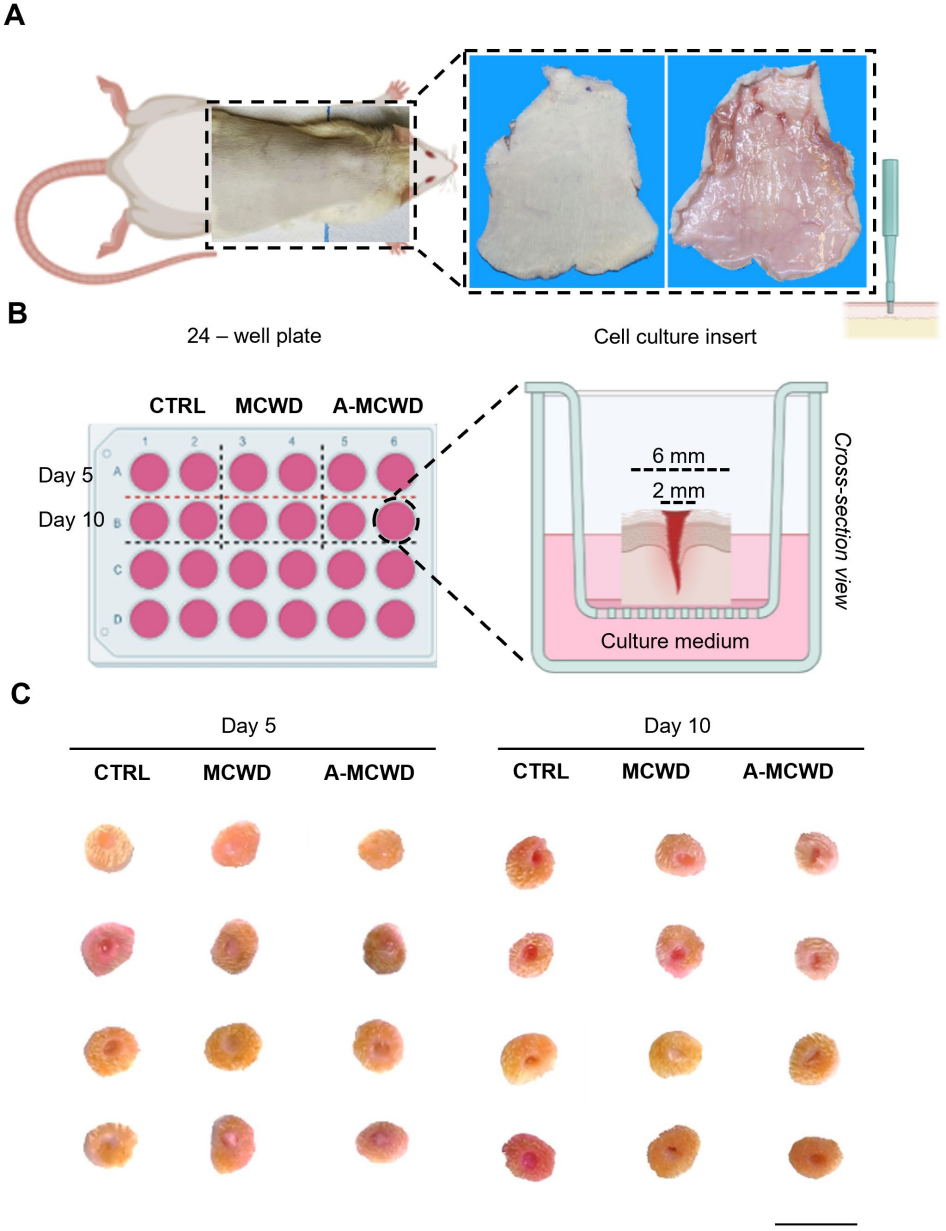
Wound Healing Organ culture (WHOC) Model: experimental set-up. **(A)** Representative images of excess hair removal and excision of dorsal skin in rats. **(B)** Scheme representing the 24 – well plate of the WHOC model used in the current study and the culture insert. Illustrations were created with BioRender.com. **(C)** Macroscopic observation of skin biopsies after 5 and 10 days of culture; scale bar = 1 cm.

In Figure 2 it is depicted the experimental set-up. The rat skin of the entire dorsum that was excised surgically to obtain explants (Figure 2A) and 56 skin discs were created using a 6 mm biopsy punch and, on each of them, a deep partial-thickness (DPT) incisional wound was performed mechanically by gently applying a 2 mm biopsy punch (Figure 2B). The skin discs were randomly assigned into three different groups (Figure 2C). After application to the skin explant, both wound dressings reduced their volume by absorbing liquids (e.g., cell culture medium), hence resulting in a more compact collagen fibrils network leading to whole coverage of the wound and maximized protection against fluid loss and bacterial infection. Explants were similar in macroscopic appearance up to day 10 and allowed to perform all analysis.

### 3.2 Histopathological observations

**Figure 3:**
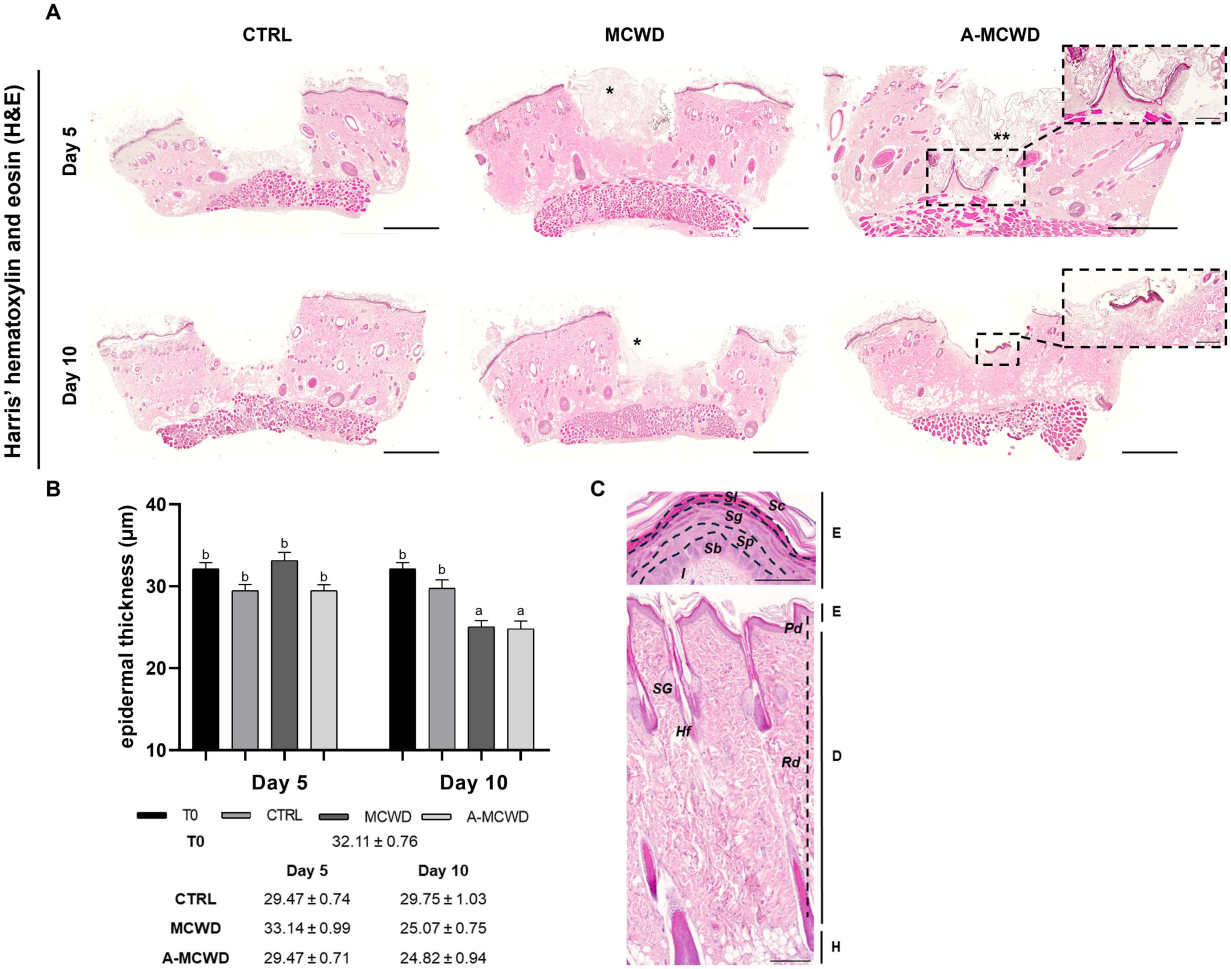
Harris’ hematoxylin and eosin (H&E). Representative skin histological microphotographs H&E-stained: comparison of untreated (CTRL), MCWD- and A-MCWD-treated wounds. **(A)** Panel of H&E-stained sections at 5 and 10 days post-wounding; scalebar = 1,000 μm, entire sample. In the dotted boxes, microphotographs of A-MCWD-treated wounds showing initial re-epithelization, magnification 50x; scale bar = 200 μm. *, marine collagen wound dressing; **, antioxidant-enriched marine collagen wound dressing. **(B)** Quantification of epidermal thickness (μm). Data are expressed as mean ± SEM. Different letters within time points means statistically significant different values for *p* < 0.05. **(C)** Representative microphotographs of rat dorsal skin and its layers (*panniculus* is missing). Above, skin epithelium, magnification 100x, scalebar = 200 μm. Below, whole skin, magnification 800x, scalebar = 50 μm. E, epithelium; D, dermis; H, hypodermis; Sc, stratum corneum; Sl, stratum lucidum; Sg, stratum granulosum; Sp, stratum spinosum; Sb, stratum basale; l, lamina propria; Pd, papillary dermis; Rd, reticular dermis; SG, sebaceous gland; Hf, hair follicle.

The effects of the different treatments at different timepoints are displayed in Figure 3A. Harris’ hematoxylin and eosin (H&E) staining revealed signs of skin regeneration at both 5 and 10 days after wounding in both treated groups. Hyperplastic epidermis (i.e., neo-epithelium) is generally encountered at the wound edges. Newly formed dermal and epidermal components are found in the wound bed in only a few samples of the A-MCWD-treated group. To assess the health status of the skin samples, the entire thickness of the epidermis was evaluated. A gradual reduction is evident comparing all samples from day 0 to day 10 and from day 5 to day 10. Moreover, differences are present between the untreated (control) and the treated groups at day 10 of culture (Figure 3B). In Figure 3C it is represented the physiological organization in layers of the rat skin of the dorsum.

**Figure 4:**
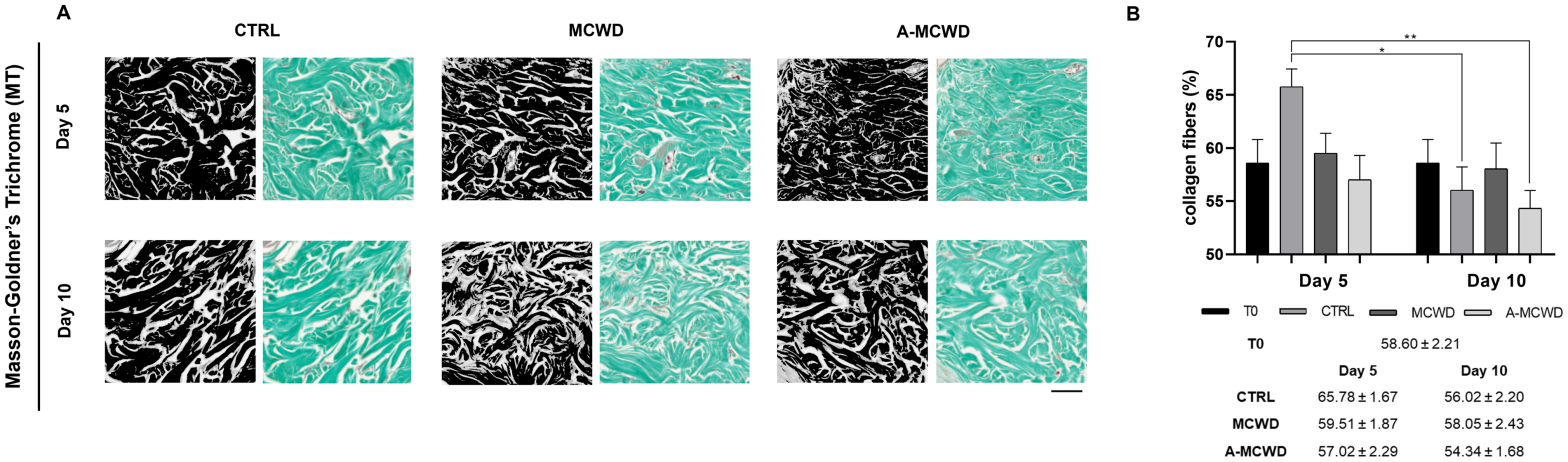
Masson-Goldner’s Trichrome (MT). Representative skin histological microphotographs MT-stained: comparison of untreated (CTRL), MCWD- and A-MCWD-treated wounds at 5 and 10 days. **(A)** Dermis regions (ROI) of 8-bit format and RGB images; scalebar = 50 μm. **(B)** Quantification of collagen fibers relative to total area (%). Data are expressed as mean ± SEM; *p < 0.05, **p < 0.01.

Histological microphotographs of Masson-Goldner’s Trichrome (MT)-stained skin biopsies were taken to assess the density of collagen fibers during the culture period. The area occupied by collagen fibers was quantified by converting RGB images to 8-bit format (Figure 4A). Generally, from day 5 to day 10, a slight decrease in the amount of light green fibers was observed, in particular in the control group (Figure 4B).

### 3.3 Immunohistochemical results

**Figure 5:**
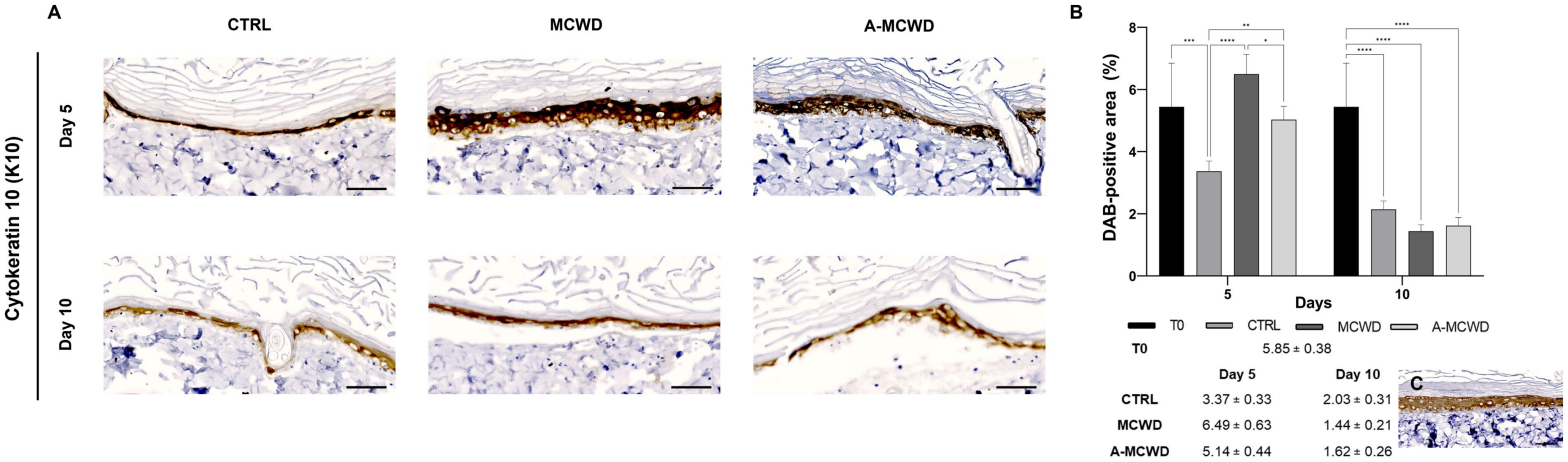
Cytokeratin 10 (K10). Representative skin immunohistochemical microphotographs of K10: comparison of untreated, MCWD- and A-MCWD-treated wounds. **(A)** Panel of immunohistochemical DAB-stained sections at 5 and 10 days; high magnification images (400x), scalebar = 50 μm. **(B)** Quantification of DAB-positive area (data are reported as percentages, %). Data are expressed as mean ± SEM; *p < 0.05, **p < 0.01, ***p < 0.001, ****p < 0.0001. **(C)** Epidermal barrier at day 0 (T0), magnification 400x, scalebar = 50 μm.

High magnification microphotographs of the epidermis showed cytokeratin 10 (K10) positivity for the suprabasal layer in all groups (Figure 5A). At day 5 of culture, A-MCWD-treated group showed a similar DAB staining intensity to that of the control at day 0 (T0), whereas in MCWD-treated wounds the immunopositivity was higher than T0 or A-MCWD (*p* = 0.0211). Conversely, control wounds at day 5 showed a significant decrease in DAB positivity compared to the other groups at the same timepoint (vs. MCWD, *p* < 0.0001; vs. A-MCWD, *p* = 0.0074). On day 10, all groups exhibited a stastically significant reduction in DAB positivity compared to samples at T0 (*p* < 0.0001). Generally, from day 5 to day 10 of culture, a decrease in DAB signal was observed in all skin explants (Figure 5B). Figure 5C shows a representative image of K10 positivity in the epidermis at day 0 (T0); this protein is expressed in early differentiated cells and in all upper differentiated layers of the epidermis, which helps to maintain epidermal homeostasis and skin barrier integrity.

**Figure 6:**
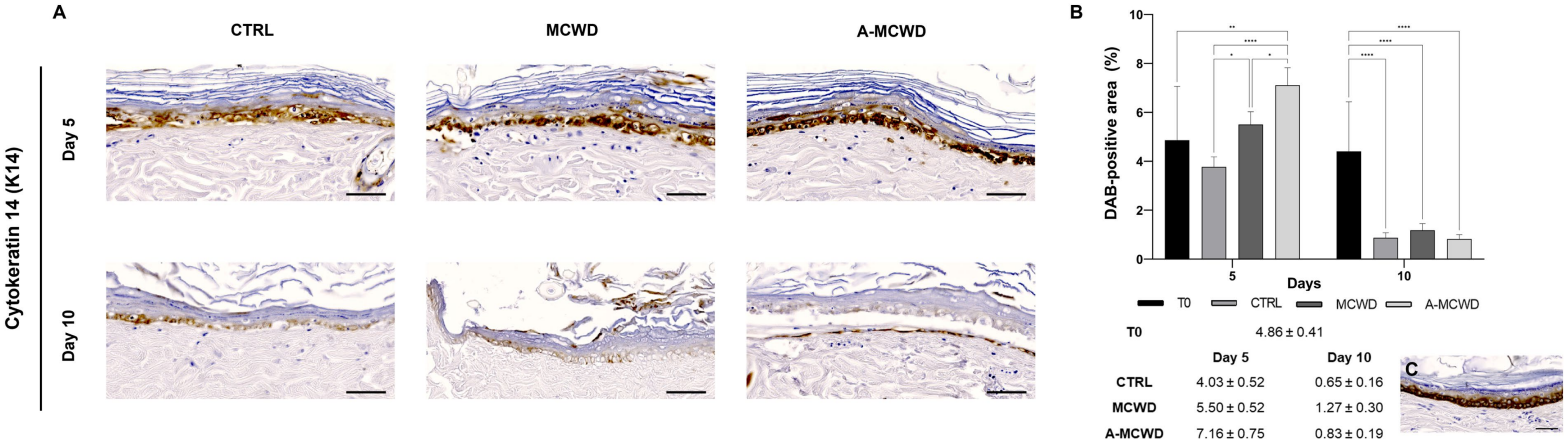
Cytokeratin 14 (K14). Representative skin immunohistochemical microphotographs of K14: comparison of untreated, MCWD- and A-MCWD-treated wounds. **(A)** Panel of immunohistochemical DAB-stained sections at 0, 5, and 10 days; high magnification images (400x), scalebar = 50 μm. **(B)** Quantification of DAB-positive area (data are reported as percentages, %). Data are expressed as mean ± SEM; *p < 0.05, **p < 0.01, ****p < 0.0001. **(C)** Epidermal barrier at day 0 (T0), magnification 400x, scalebar = 50 μm.

High magnification images of the epidermis showed cytokeratin 14 (K14) positivity for the basal layer in all samples (Figure 6A). As the culture time increases, a significant decrease in DAB positivity was evidenced. As depicted in Figure 6B, on day 5 of culture MCWD group showed similar DAB positivity compared to the unwounded skin at day 0 (T0) while A-MCWD presented a higher immunolabeling compared to T0 (*p* = 0.0017); on the other hand, the control group wounds showed a lower positivity for K14 compared to MCWD (*p* = 0.0244) and A-MCWD (*p* < 0.0001). Furthermore, A-MCWD-treated wounds presented with a significantly higher immunoreactivity for K14 compared to MCWD-treated lesions (*p* = 0.0380). On the contrary, a significant decrease was detected on day 10 in all skin explants compared to day 0 (*p* < 0.0001). In Figure 6C the epidermis at day 0 (T0) is shown, immunolabeling can be appreciated in cells of the basal layer (undifferentiated).

**Figure 7:**
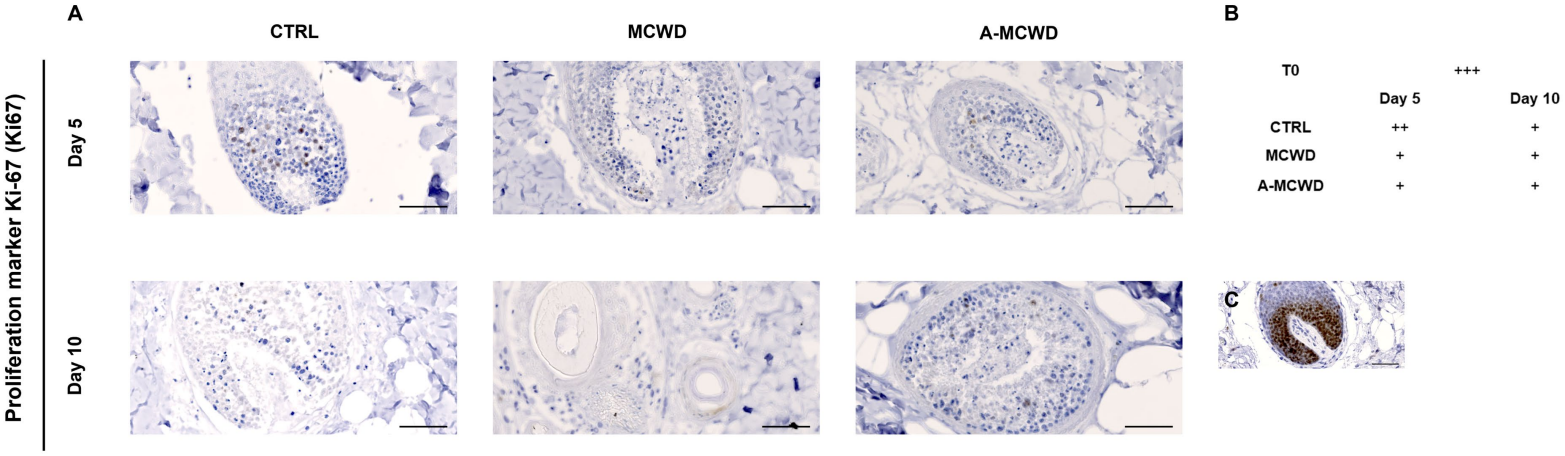
Proliferation marker protein Ki-67 (Ki67). Representative skin immunohistochemical microphotographs of Ki67. **(A)** Panel of Ki67 DAB-stained skin appendages of untreated, MCWD- and A-MCWD-treated wounds at 5 and 10 days, magnification 400x, scalebar = 50 μm. **(B)** Ki67 score (manual) was evaluated as: negative (0), weak (+), moderate (++), strong (+++). **(C)** Skin appendages at day 0 (T0), magnification 400x, scalebar = 50 μm.

In Figure 7A, microphotographs of skin appendages labeled for proliferation marker protein Ki-67 (Ki67) are shown. Ki67 positivity was assessed scoring from 0 (negative) to +++ (strong). A reduction of Ki67 positivity occurred during the culture period in all groups (Figure 7B). In Figure 7C it is depicted a representative image of cells immunolabeled by Ki67 in the hair follicle at day 0 (T0).

### 3.4 Gene expression analysis

**Figure 8:**
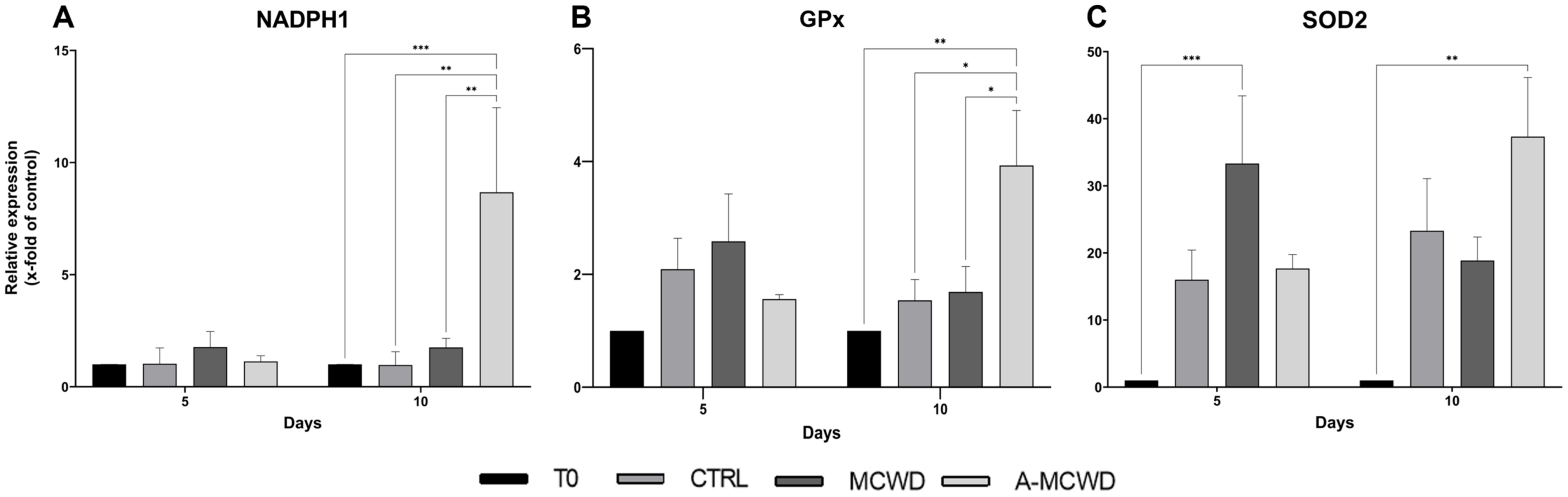
Gene expression analysis of NADPH Oxidase 1, Glutathione Peroxidase 1 and Superoxide Dismutase 2. The mRNA levels of **(A)** NADPH Oxidase 1 (NADPH1), **(B)** Glutathione Peroxidase 1 (GPx1) and **(C)** Superoxide Dismutase 2 (SOD2) at 0, 5 and 10 days after wounding. Relative gene expression levels were normalized using two reference genes (TBP and RPLP0) and uninjured skin was used as the calibrator sample. Data are expressed as mean ± SEM; *p < 0.05, **p < 0.01, ***p < 0.001.

RT-PCR analysis revealed that NADPH1 reached the highest gene expression levels in the A-MCWD-treated group. Particularly, A-MCWD at day 10 NADPH1 was 8-fold higher compared to both T0 (A-MCWD 8.68 ± 3.08 vs. T0 1, *p* = 0.006) and control wounds at day 10 (A-MCWD Day 10 8.68 ± 3.08 vs. CTRL Day 10 0.97 ± 0.48, *p* = 0.0013), while it was 4-fold higher than MCWD at day 10 (A-MCWD 8.68 ± 3.08 vs. MCWD 1.75 ± 0.35, *p* = 0.0019). Overall, both untreated and MCWD-treated groups maintained basal gene expression levels similar to the untreated group (T0) (Figure 8A). Likewise, GPx1 reached maximum expression after 10 days of culture in the A-MCWD-treated group, which was 3-fold higher than T0 (A-MCWD 3.93 ± 0.80 vs. T0 1, *p* = 0.0033) and 2-fold higher than both the control (A-MCWD Day 10 3.93 ± 0.80 vs. CTRL 1.54 ± 0.30, *p* = 0.0243) and MCWD groups at the same timepoint (A-MCWD 3.93 ± 0.80 vs. MCWD 1.69 ± 0.39, *p* = 0.0283). As in the case of NADPH1, both the untreated and the MCWD-treated groups maintained basal levels of GPx1 expression after 10 days of culture (Figure 8B). Conversely, gene expression of Superoxide Dismutase 2 (SOD2) was significantly different in both the treated groups compared to T0 at day 5 for MCWD (vs. MCWD 33.33 ± 8.73, *p* = 0.0961) and day 10 for A-MCWD (vs. A-MCWD 37.33 ± 6.23, *p* = 0.0023) (Figure 8C).

**Figure 9:**
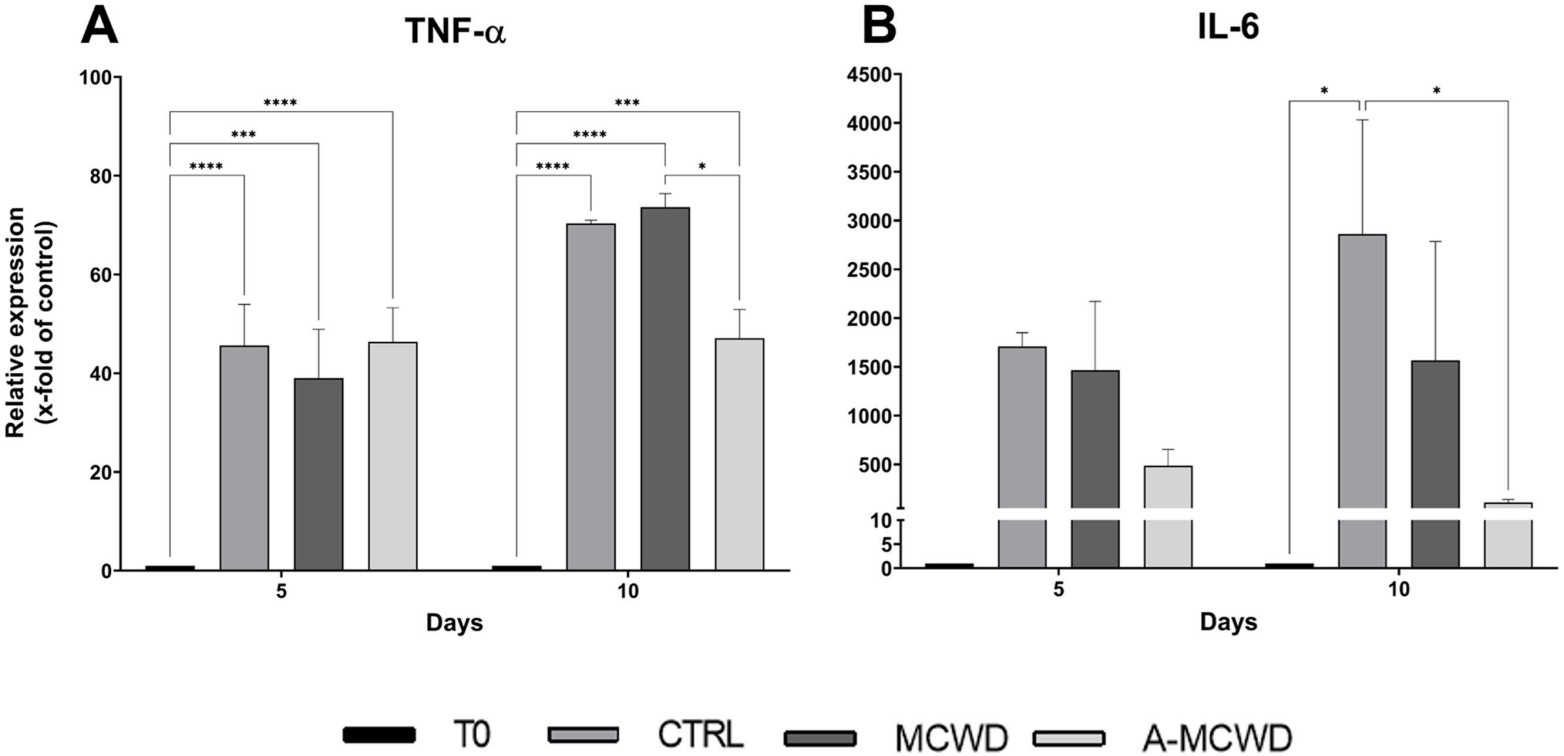
Gene expression analysis of Tumor Necrosis Factor alpha and Interleukin 6. The mRNA levels of **(A)** Tumor Necrosis Factor (TNF-α) and **(B)** Interleukin 6 (IL-6) at 0, 5 and 10 days after wounding. Relative gene expression levels were normalized using two reference genes (TBP and RPLP0) and uninjured skin was used as the calibrator sample. Data are expressed as mean ± SEM; *p < 0.05, ***p < 0.001, ****p < 0.0001.

In addition, increased expression of TNF-*α* was evident after 5 days of culture in all the groups compared with T0 (T0 1 vs. CTRL 45.62 ± 7.21, *p* < 0.0001; MCWD 39.02 ± 8.56, *p* = 0.0006; A-MCWD 46.34 ± 5.98, *p* < 0.0001). Interestingly, TNF-α levels further increased up to 10 days of culture in all the groups except in the A-MCWD-treated group. Indeed, at day 10, the gene expression of TNF-α was slightly lower in the A-MCWD- group compared to MCWD-treated group (A-MCWD 47.05 ± 4.79 vs. MCWD 73.65 ± 2.36, *p* = 0.0281) (Figure 9A). Similarly, IL-6 gene expression was lower in the A-MCWD-treated groups compared to CTRL, showing a 3-fold reduction in the A-MCWD-treated samples on day 5 (CTRL 1707.35 ± 115.95 vs. A-MCWD 487.01 ± 144.08) but the difference is not statistically significant; however a 25-fold reduction of mRNA levels was observed in A-MCWD-treated leasions after 10 days (CTRL 2859.03 ± 1015.14 vs. A-MCWD 110.18 ± 26.45, *p* = 0.0360) (Figure 9B).

**Figure 10:**
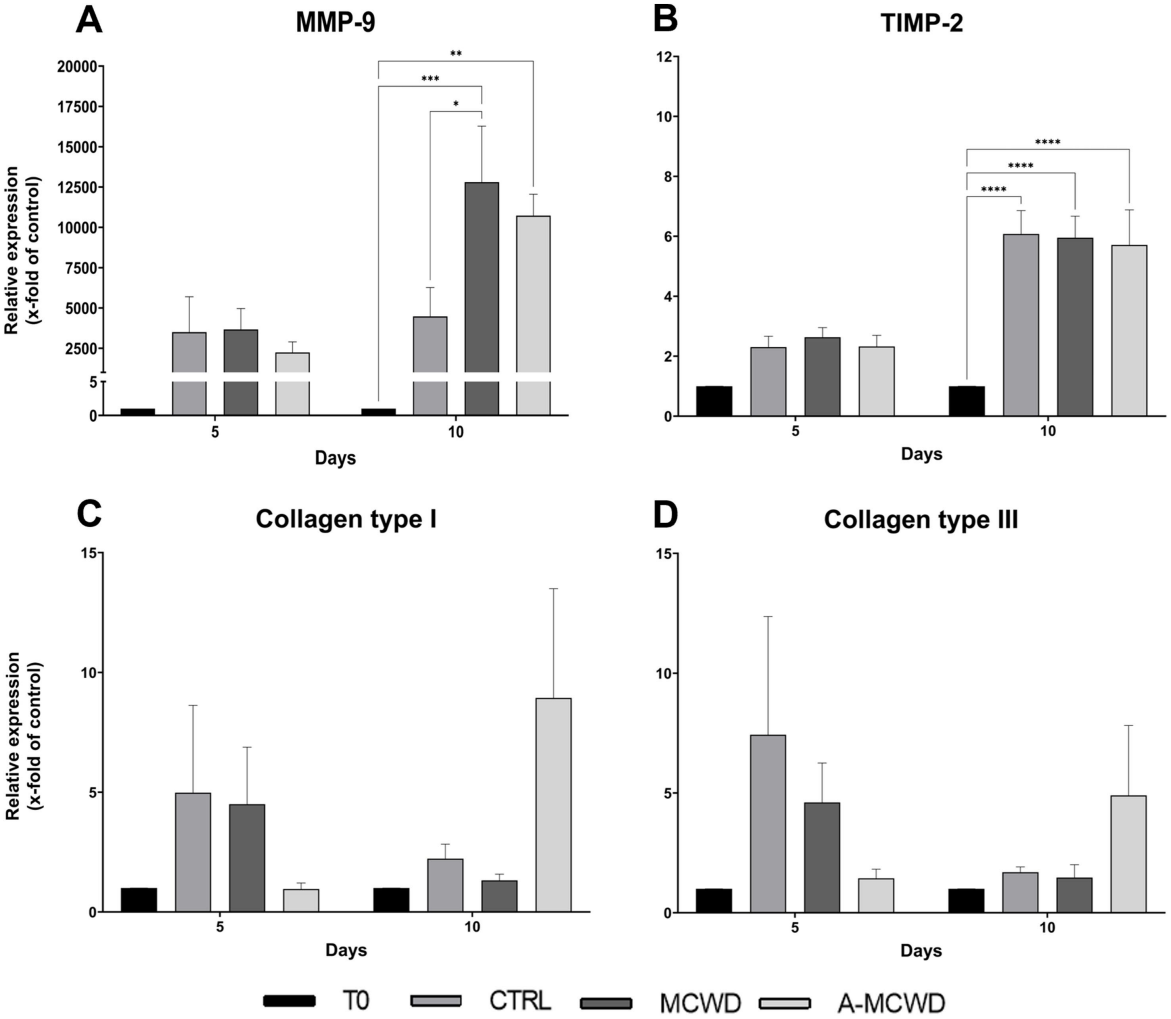
Gene expression analysis of Matrix Metalloproteinase 9, Tissue Inhibitor of Metalloproteinase 2, Collagen type I and Collagen type III. The mRNA levels of **(A)** Matrix Metalloproteinase 9 (MMP-9), **(B)** Tissue Inhibitor of Metalloproteinase 2 (TIMP-2), **(C)** Collagen type I (COL1A1) and of **(D)** Collagen type III (COL3A1) at 0, 5 and 10 days after wounding. Relative gene expression levels were normalized using two reference genes (TBP and RPLP0) and uninjured skin was used as the calibrator sample. Data are expressed as mean ± SEM; *p < 0.05, **p < 0.01, ***p < 0.001, ****p < 0.0001.

Further, after 10 days, MMP-9 gene expression increased compared to control wounds with the application of the MCWD in a significant way (CTRL 4477.60 ± 1548.68 vs. MCWD 12804.10 ± 3006.14, *p* = 0.0117) and with A-MCWD (CTRL 4477.60 ± 1548.68 vs. A-MCWD 10722.35 ± 1150.49). Notably, such an increase was also observed when treated samples were compared with uninjured skin. In this case, in both MCWD- and A-MCWD-treated samples after 10 days of culture, MMP-9 transcripts were more than 10,000 times higher compared to T0 in both treated groups (T0 1 vs. MCWD 12804.10 ± 3006.14, *p* = 0.0001; A-MCWD 10722.35 ± 1150.49, *p* = 0.0011) (Figure 10A). In contrast, the relative expression of TIMP-2 was significantly higher after 10 days of culture regardless of treatment or non-treatment when compared to T0 (T0 1 vs. CTRL 6.08 ± 0.68, ****p < 0.0001; MCWD 5.96 ± 0.62, ****p < 0.0001; A-MCWD 5.71 ± 0.95, ****p < 0.0001) (Figure 10B).

Differently, gene expression of both COL1A1 and COL3A1 did not show any significant difference among groups at the same timepoints. However, an increasing trend of gene expression was noticed in the A-MCWD-treated group compared to other groups at day 10. In fact, in A-MCWD-treated samples, COL1A1 was 4- and 6-fold higher with respect to control wounds (CTRL 2.23 ± 0.52 vs. A-MCWD 8.93 ± 3.95) and MCWD-treated samples at the same timepoint (MCWD 1.32 ± 0.23 vs. A-MCWD 8.93 ± 3.95), respectively (Figure 10C). A similar trend was observed for COL3A1 gene expression, with a delayed transcription of the gene after 10 days of treatment with A-MCWD (Figure 10D).

**Figure 11:**
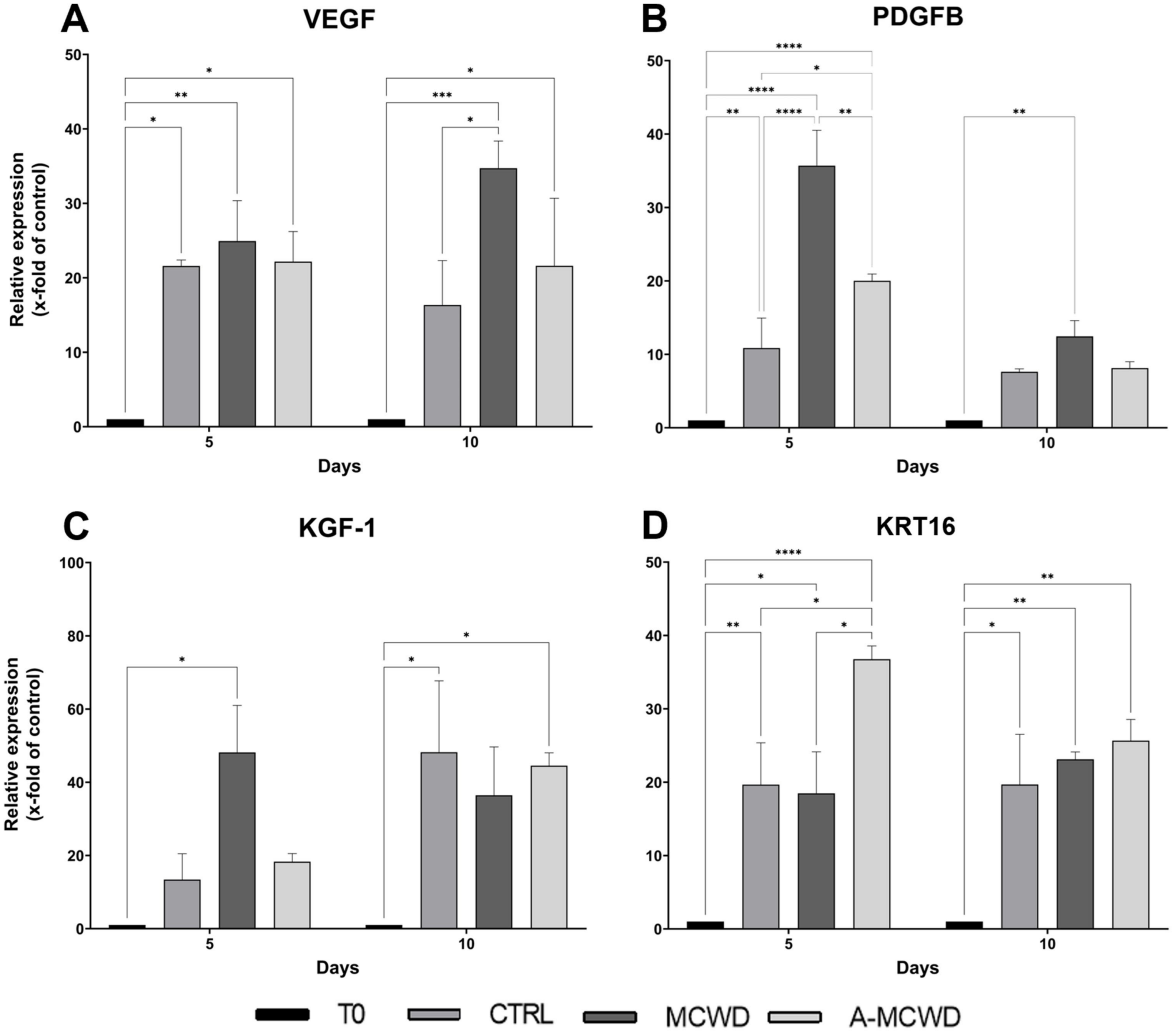
Gene expression analysis of Vascular Endothelial Growth Factor, Platelet Derived Growth Factor Subunit B, Keratinocyte Growth Factor 1 and Keratin 16. The mRNA levels of **(A)** Vascular Endothelial Growth Factor (VEGF), **(B)** Platelet Derived Growth Factor Subunit B (PDGFB), **(C)** Keratinocyte Growth Factor (KGF-1) and **(D)** Keratin 16 (KRT16) at 0, 5 and 10 days after wounding in untreated, MCWD- and A-MCWD-treated wounds. Relative gene expression levels were normalized using two reference genes (TBP and RPLP0) and uninjured skin was used as the calibrator sample. Data are expressed as mean ± SEM; *p < 0.05, **p < 0.01, ***p < 0.001, ***p < 0.0001.

Notably, VEGF gene expression was increased at day 5 in all the samples compared to T0 (T0 1 vs. CTRL 21.59 ± 0.70, *p* = 0.0241; MCWD 24.93 ± 4.70, *p* = 0.0075; A-MCWD 22.18 ± 3.50, *p* = 0.0197). However, after 10 days of culture, the MCWD-treated samples displayed a further increase of VEGF expression when compared with T0 (T0 1 vs. MCWD 34.73 ± 3.15, *p* = 0.0002). In addition, in A-MCWD-treated group, VEGF transcript was significantly higher compared to the control group at day 10 (A-MCWD 21.62 ± 7.85 vs. CTRL 16.34 ± 5.17, *p* = 0.0239), in which the expression was 2-fold lower (Figure 11A). Interestingly, the gene expression of PDGFB was significantly increased after 5 days of culture in both the MCWD- and A-MCWD-treated groups compared to control lesions (CTRL 10.87 ± 3.54 vs. MCWD 35.68 ± 3.42, *p* < 0.0001; A-MCWD 20.02 ± 0.76, *p* = 0.0272), and, notably, differences between MCWD- and A-MCWD-treated samples were observed at the same timepoint (MCWD 35.68 ± 3.42 vs. A-MCWD 20.02 ± 0.76, *p* = 0.0013) (Figure 11B).

KGF-1 showed a greater expression in the MCWD-treated group at day 5 of culture compared to day 0 (MCWD 48.17 ± 11.11 vs. T0 1, *p* = 0.0128). A 3-fold higher expression was also observed compared to the control group (MCWD 48.17 ± 11.11 vs. CTRL 13.41 ± 6.12) whereas a 2-fold higher expression was evidenced in comparison to the A-MCWD-treated samples at the same timepoint (MCWD 48.17 ± 11.11 vs. A-MCWD 18.29 ± 1.94). Noteworthy, by day 10 of culture, KGF-1 expression reached the same values in cultured skin explants, with a significant higher gene expression compared to day 0 (T0 1 vs. CTRL 48.23 ± 16.87, *p* = 0.0127; MCWD 36.44 ± 11.44; A-MCWD 44.54 ± 3.03, *p* = 0.0232) (Figure 11C).

Finally, KRT16 gene expression was significantly increased in the A-MCWD-treated group after 5 days of culture compared with all the other groups (A-MCWD 36.76 ± 1.57 vs. CTRL 19.67 ± 4.94, *p* = 0.0179; MCWD 18.48 ± 4.90, *p* = 0.108) (Figure 11D).

## 4 Discussion

Skin wound management is a significant challenge in veterinary practice as traditional therapies often result in poor outcomes, which can also lead to substantial economic costs. An increasing number of novel skin regenerative therapies is currently under investigation (40, 41, 54). This study contributes to the advancement of knowledge on circular economy and blue biotechnology approaches in skin regenerative medicine and tissue engineering. We previously demonstrated that some sea food wastes, namely sea urchins, can be profitably used to obtain high added value marine-derived antioxidant molecules (the so called polyhydroxynaphtoquinones, PHNQs, a class of potent polyphenols typical of sea urchins(42, 43)) and collagen (37–39) with the latter being efficiently used to produce collagen-based biomaterials for tissue regeneration (39–41). Sea urchin-derived collagen-based wound dressings (CBWDs), namely “first generation” marine collagen wound dressing (MCWD, made of sole collagen), and novel “second generation” antioxidant-enriched marine collagen wound dressing (A-MCWD, made of collagen combined with PHNQs), were already characterized in Martinelli et al. (2024, submitted, under review). Characterization tests prior to *ex vivo* testing (such as swelling test and degradation kinetics evaluation) have shown that when PHNQs are added to MCWD, they increase the stability of the biomaterials (as shown in the Supplementary Table S2). As a step forward, in this work we tested the efficacy of both collagen-based and marine-derived dressings, the MCWD and the A-MCWD, for the treatment of skin wounds in an *ex vivo* preclinical model.

CBWDs were applied to *ex vivo* induced injuries and their efficacy was compared and evaluated. More specifically, after 5 and 10 days of culture, skin explants were tested for factors involved in re-epithelialization, inflammation, and antioxidant activity.

In cutaneous injuries, proliferation and migration of keratinocytes are fundamental steps of re-epithelialization, which may occur either from wound edges or from any transected hair follicles and sweat gland ducts (52, 53). Although repair is a physiological process, dressings are used to promote faster re-epithelization of extensive lesions. Thus, after application of the novel marine CBWDs, epidermal growth was assessed. Harris’ hematoxylin and eosin (H&E) as well as Masson-Goldner’s Trichrome (MT) staining, showed epidermal regeneration, characterized by hyperplastic epidermis 5 days after wounding in samples treated with the A-MCWD. Additionally, the A-MCWD-treated group displayed the formation of new skin with reorganized ECM within the wound bed. On the contrary, no signs of skin development were observed in the wounds of untreated or MCWD-treated samples, suggesting an exclusive beneficial effect of the antioxidant-enriched wound dressing. To further investigate this process, immunohistochemical and gene expression analysis were carried out. Cytokeratin 10 (K10), a key protein for epidermal growth, maturation, and homeostasis (55), and Cytokeratin 14 (K14), a marker of basal (germinal) layer progenitor cells (56, 57), resulted more expressed in both CBWDs groups than in the control suggesting a supportive role of both biomaterials in promoting keratinocytes differentiation and maturation. Concomitantly, the gene expression of keratinocyte growth factor-1 (KGF-1), also known as fibroblast growth factor 7 (FGF-7), was higher in wounds treated with CBWDs. The obtained results indicate that both MCWD and A-MCWD may act as promoters of keratinocytes activation, which early onset was predicted by a peak in the gene expression of KGF-1 at day 5 post-treatment. Indeed, KGF-1 is a paracrine growth factor which expression increases massively in various mesenchymal cells, predominantly fibroblasts, during the early stages of wound healing, specifically and positively regulating the proliferation and migration of keratinocytes and thus facilitating the re-epithelialization process (58, 59). In a similar manner, keratin 16 (KRT16) is usually rapidly induced in keratinocytes after wounding (60). As an activation- and hyperproliferation-associated keratin, its early expression after cutaneous injuries is fundamental for the correct progression of inflammation, keratinocyte migration and epidermal hyperplasia (61–63). Especially at day 5, as observed for KGF, the application of the A-MCWD biomaterial enhanced the expression of KRT16 compared to other groups.

It is well known that ROS are able to orchestrate the initial phases of wound repair by inducing the recruitment of immune cells, fibroblasts, epithelial and endothelial cells (64), ultimately promoting angiogenesis, and removing potential pathogens. Despite the healing-enhancing role of ROS at the injury site, the maintenance of their balance is critical in determining a physiological healing response (65). As a therapeutic strategy, the manipulation of the oxidant-antioxidant system controlling ROS levels indirectly by using bioactive compounds of marine origin (i.e., PHNQs) has already proven effective (43, 66, 67). Indeed, PHNQs contain several hydroxyl groups appropriate for free-radical scavenging, which diminish ROS levels and prevent redox imbalance, suggesting their potential use for several different medical conditions characterized by a consistent oxidative stress (e.g., diabetes, cardiovascular diseases, etc.) (68). In the present work, the effects of sea urchin-derived biocomposites on the expression of NADPH oxidase (NADPH1), glutathione peroxidase 1 (GPx1) and superoxide dismutase 2 (SOD2) were investigated. NADPH oxidase enzyme complexes (NOX) are the main producer of endogenous ROS. Specifically, NOX enzymes are responsible for reducing oxygen to superoxide radical (O_2_^▪−^), which boosts the formation of further ROS in the cellular and extracellular spaces. Superoxide radical is then converted by superoxide dismutase (SOD) to hydrogen peroxide (H_2_O_2_) (69), a weak oxidizing agent that promotes the biosynthesis of pro-inflammatory cytokines, such as tumor necrosis factor-*α* (TNF-α) and interleukin 6 (IL-6), inducing the migration of phagocytic cells to the site of injury (70–72). Eventually, H_2_O_2_ is reduced to water by enzymes of the GPx family (73). In this experimental model, after 10 days of treatment, A-MCWD significantly increased NADPH1 mRNA levels by 8- and 4-fold, compared with untreated and MCWD-treated wounds, respectively. Also, 10 days after wounding, A-MCWD significantly increases transcription of GPx1 gene (by 2-fold, compared to both control and MCWD-treated groups). Concomitantly, A-MCWD-treated samples showed reduced gene expression of TNF-α and IL-6, being the latter 25-fold lower than control group and 14-fold lower than MCWD-treated wounds. Since enhanced and prolonged TNF-α gene expression leads to impaired healing (74) and elevated IL-6 levels have been found in a variety of chronic inflammatory disorders characterized by fibrosis (75), we assume that the addition of PHNQs to the CBWD might play a role in counteracting and preventing excessive production of pro-inflammatory factors that occurs locally in the *ex vivo* model. As a matter of fact, previous works have demonstrated that excessive ROS levels are able to modulate inflammation (76–79) by regulating different cell signaling pathways such as those of NF-κB (80), MAPK (77), PI3K/AKT (81), or JAK/STAT (82); doing so, the downstream effects are known to be related to the up-regulation of the expression of pro-inflammatory cytokines and chemokines, as long as shifting macrophages polarization toward the M1 pro-inflammatory phenotype, hence hastening the inflammatory response in the wound (83). For this reason, a scavenging biomaterial would be ideal in restoring ROS homeostasis by favoring free radicals clearance and consequently limiting excessive inflammation by indirectly reducing inflammatory factors.

Recruitment of keratinocytes but also fibroblasts and endothelial cells orchestrate wound closure and angiogenesis. During these processes, growth factors such as platelet-derived growth factor B (PDGFB), vascular endothelial growth factor (VEGF), and fibroblast growth factor 7 (FGF-7 or KGF-1) (84) are released, promoting matrix turnover, fibroplasia, angiogenesis, and re-epithelialization. PDGFB directs fibroblast to a pro-fibrotic phenotype, leading to deposition of ECM proteins (85). Fibroblasts are critical actors in the wound repairing process as they contribute to collagen synthesis, fundamental in restoring and filling the wound gap (64). Noteworthy, RT-PCR analysis revealed that both MCWD and A-MCWD greatly induce PDGFB transcription in the early phase of healing. Indeed, in the MCWD-treated group, PDGFB gene expression was 3-fold higher than control and 1-fold higher than A-MCWD-treated samples. On the other hand, no significant differences were observed in the expression of collagens genes. It should be noted that keratinocytes interact with the ECM structural proteins via integrin receptors and migrate through the damaged tissue into the wound bed, where re-epithelialization occurs. Matrix metalloproteinases (MMPs), particularly MMP-9, facilitate integrin receptor dissociation, being therefore essential for keratinocyte migration (85). For normal wound healing, a balance of MMPs and their inhibitors (i.e., tissue inhibitors of metalloproteinases, TIMPs) in the affected tissues is required, as their enzyme activity greatly influences skin regeneration, and a possible imbalance could lead to wound chronicity or excessive scarring processes (64, 86). MMP-2 and -9 play an active role in wound repair by degrading a wide range of extracellular proteins, which ultimately release numerous biological factors involved in the healing process (84, 87). MMP-2 (gelatinase A, type IV collagenase) and MMP-9 (gelatinase B, type IV collagenase) are effective in digesting damaged fibrillar collagens (gelatin) type I, III, and type IV [main component of the basement membranes (88)], respectively (89, 90). MMP-2 is often expressed in a constitutive manner whereas MMP-9 transcription is modulated by a multitude of physiological stimuli (cytokines, chemokines, and growth factors) and by ROS (91). Tissue inhibitors of metalloproteinases 1 and 2 (TIMP-1/TIMP-2) are the corresponding tissue inhibitors of MMP-9 and MMP-2 in tissues. Considering that MMPs and TIMPs exert their activity in both physiological (i.e., tissue repair) and pathological processes (i.e., tumorigenesis), their concentrations must be highly regulated (64, 86). Consistently, our results revealed that the application of CBWDs significantly increased MMP-9 transcription, which was a 2-fold higher in MCWD- and A-MCWD-treated samples than in control group after 10 days of treatment, without major influence on TIMP-2 mRNA levels.

Overall, based on the obtained results both marine collagen-based wound dressing might play different roles in wound management based on the nature and etiology of the wound. Both biomaterials possess typical wound dressings characteristics that can sustain and promote wound healing and might be ideal to treat acute fresh wounds acting as a valid alternative to already commercially available products (39–41). However, the added value of the A-MCWD, which is the addition of PHNQs and their scavenging properties, might give it the opportunity to be ideal for the treatment of chronic wounds or hard-to-heal ulcers characterized by a consistent and excessive inflammatory component such as diabetic foot ulcers, venous leg ulcers, or pressure ulcers (92, 93). Hence, the application of a scavenging biomaterial such as the A-MCWD might contribute to the physiological progression of the wound healing process toward a resolution of the inflammatory phase by restoring ROS homeostasis in the wound bed (73, 76–79).

Effective experimental strategies for understanding skin regeneration processes are highly demanded and *in vitro*/*ex vivo* wound healing models might provide the answer and be a valid alternative to animal studies (94). To date, animal models are still an important and compulsory component for regulatory approval and pre-clinical screening of new drugs (95). However, given the growing ethical concern about the use of sentient creatures for research purposes, efforts have been recently directed toward the practical implementation of the three R’s principle, i.e., reduction, refine, and replacement of animals (96). In this perspective, *ex vivo* organ culture (EVOC) is being considered a useful preclinical model to evaluate skin-recovery agents, as well as an alternative experimental approaches to minimize the use of animals for repair studies while maintaining access to an environment that replicates natural tissues and supports a variety of cell types (97). Nevertheless, the EVOC system is limited by the lack of adequate skin innervation and blood perfusion, which prevents adequate healing and precludes long-term organ cultivation. There are growing evidences that the cutaneous nervous system regulates physiological and pathophysiological pathways, including cell growth and differentiation, immunity, inflammation, and all phases of tissue repair. Indeed, peripheral nerves play an important and complex role in skin wound healing by releasing neuromediators such as neuropeptides, neurohormones, cytokines, and growth factors involved in the tissue and cell healing process, both in the wound area (e.g., catecholamines, acetylcholine, and neuropeptides modulate immune activity, re-epithelization, and wound contraction (98) and in the intact skin near the wound (maintenance of skin homeostasis) (99)). Consequently, the lack of innervation and blood supply, and thus of oxygen and inflammatory cells, could have a significant, though not fully understood, impact on wound healing and skin organ maintenance *in vitro*. One of the main drawbacks of skin culture is precisely the relatively short timeframe in which wound healing can be studied, as there is a chance that the effects of tissue deterioration might bias the final results (100). In fact, we have observed that a slow modification in skin microarchitecture occurs as the culture progressed, resulting in a slight reduction in epidermal thickness and amount of collagen fibers. Likewise, prominent features of skin decay are the decrease in keratin expression and the loss of proliferating cells, both along the epidermis and in secondary skin structures, which may be related to the insufficient perfusion of the *ex vivo* tissue in the upper layers (101, 102). Despite the limitations of the *ex vivo* model, in our experimental setup, no serious tissue changes occurred up to 10 days. For this reason, the model could be ideal for studying the early stages of wound healing. Therefore, also considering the unavailability of a standardized protocol for *ex vivo* skin wound healing, the suggested wound healing organ culture (WHOC) was used to test the application of clinically relevant biomedical tools and explore their regenerative potential. Prospects include the development of a more stable skin WHOC model, which will allow all stages of the wound healing process to be evaluated. In this regard, a shift from a static culture to a dynamic culture might be ideal and effective.

## 5 Conclusion

In summary, this study presents an organ *ex vivo* culture of rodent wounded skin, which might offer a useful preclinical model to study the process of skin repair and evaluate potential therapeutic agents. The effect of sea urchin waste-derived CBWDs on wound healing in rat skin was studied *ex vivo* for the first time. In general preliminary data suggest that these ecofriendly and marine CBWDs can be a valuable tool to maintain a moist environment, accelerating epidermal cell migration and thereby facilitating wound closure. Particularly, biomaterials composed by a combination of collagen and antioxidants (PHNQs) appear as a promising dressing for skin regeneration by reducing inflammation and promoting the appearance of dermal and epidermal components within the wound bed. Considering the encouraging results of this *ex vivo* pilot study, next studies should focus on testing the biocompatibility and regeneration efficacy of these biomaterials in a more complex model, such as in *in vivo* test. For this reason, advancing research in the clinical field with a substantial number of samples may help to understand the actual therapeutic of MCWD and A-MCWD role in skin wound repair. In perspective, a further generation of sea urchins collagen-based scaffolds are under development by 3D printing. This will allow to produce patient-tailored biomaterials with improved mechanical properties. Challenges of these approaches will be in preserving the native fibrillar structure of sea urchin collagen Once this functional issues will be addressed, the main challenge will lie in the scaling up of the production process of these wound dressing in terms of waste abundance and recovery, industrial processing and health regulation prior to marketability. Sea urchin market annually accounts for slightly over 60,000 tons worldwide (103) which, although not comparable to the market of other animal collagen/antioxidants sources (e.g., bovine, equine, fish) it still represents a considerable rough material source (=waste). To have a clear picture of the possible scalability one has to consider that from the waste of a single sea urchin a standard wound dressing (1 cm diameter, 5 mg collagen) can be obtained. Moreover, comparing to other sourced, collagen obtained from sea urchin waste has relevant advantages in term of preserved structural integrity, including its glycosaminoglycan decoration, and absence of specific disease transmission issues or religious/cultural constrain (38, 39). Nevertheless, a critical issue for future industrial production will be the reconstruction of a full and constant supply chain connecting the different stakeholders, from sea urchin processing companies to biotech companies. According to Zilia and colleagues (103, 104) a proper sea urchin waste management and valorization toward added-value products (e.g., collagen-based wound dressing) will likely produce environmental (reduction of solid waste disposement), economic (novel markets) and social (novel job opportunities) benefits although possible drawback can be envisaged (constant supply of waste, high-cost distribution channels). Last but important, challenges will lie also in meeting the regulatory requirements of medical device development, such as monitoring and controlling the production chain to avoid any material contamination (e.g., pest control, monitoring and controlling the work environment, laboratory cleaning, monitoring and maintaining environmental conditions, rules of conduct and introduction of materials into the clean room).

## Data Availability

The original contributions presented in the study are included in the article/Supplementary material, further inquiries can be directed to the corresponding author.
