# Supplementary material for: Sea food by-products valorization for biomedical applications: evaluation of their wound regeneration capabilities in an Ex vivo skin model
Source: Front Vet Sci. 2024 Nov 18;11:1491385. doi: 10.3389/fvets.2024.1491385 (PMC11629400; doi:10.3389/fvets.2024.1491385)
Supplement: Supplementary file 1 [file Data_Sheet_1.PDF]

Target gene	Primer sequence (5'→3')
**COL1A1**	For 5'-ATCAGCCCAAACCCCAAGGAGA-3'<br>Rev 5'-CGCAGGAAGGTCAGCTGGATAG-3'
**COL3A1**	For 5'-TGATGGGATCCAATGAGGGAGA-3'<br>Rev 5'-GAGTCTCATGGCCTTGCGTGTTT-3'
**GPx1**	For 5'-AGTTCGGACATCAGGAGAATGGCA-3'<br>Rev 5'-TCACCATTCACCTCGCACTTCTCA-3'
**IL-6**	For 5'-CACTTCACAAGTCGGAGGCT-3'<br>Rev 5'-TCTGACAGTGCATCATCGCT-3'
**KGF-1 or FGF7**	For 5'-TGGCAGTTGGAATTGTGG-3'<br>Rev 5'-ATGCAGAGGTGTTGTAATGGT-3'
**KRT16**	For 5'-ATCGTTAGAGCCAAGCAGGA-3'<br>Rev 5'-TTCCACTAACTGACGCAAGG-3'
**MMP-9**	For 5'-CAAGGATGGTCTACTGGCACACG-3'<br>Rev 5'-AGGTGAAGGGAAAGTGACATGGG-3'
**NADPH1 or NOX1**	For 5'-TGGTGATCCTGATTCCTGTG-3'<br>Rev 5'-CCAGCTTATGGAAGGTGAGG-3'
**PDGFB**	For 5'-TGGAGTCGAGTCGGAAAGCT-3'<br>Rev 5'-GAAGTTGGCATTGGTGCGAT-3'
**RPL0**	For 5'-TGCTGGACATCACAGAGCAG-3'<br>Rev 5'-GATGGAGTGAGGCACTGAGG-3'
**SOD2**	For 5'-TTGGCTTCAATAAGGAGCAAG-3'<br>Rev 5'-ACACATCAATCCCCAGCAGT-3'
**TBP**	For 5'-GAGTCATGGACCAGAACAACA-3'<br>Rev 5'-TCTGAACAGGCTGTGGAGTAAG-3'
**TNF-α or TNF**	For 5'-AGCCTCTTCTCATTCCTGCTC-3'<br>Rev 5'-GTTTGCTACGACGTGGGCTAC-3'
**TIMP-2**	For 5'-CTGCGAGTGCAAGATCACA-3'<br>Rev 5'-CATCCAGAGGCACTCATCC-3'
**VEGF**	For 5'-ATCATGCGGATCAAACCTCACC-3'<br>Rev 5'-GGTCTGCATTCACATCTGCTATGC-3'

Table S1. Primer sequences used for Real Time PCR. For, forward; Rev, reverse.

	MCWD	A-MCWD	*p value*
**Area variation (%)**	39.08 ± 0.15	1.93 ± 0.07	**$p < 0.001$**
**Thickness variation (%)**	-73 ± 0.08	-48.92 ± 0.06	**$p < 0.01$**

Table S2: Area and thickness variation (%) of MCWD and A-MCWD. All data are expressed as mean ± SD. Significant differences are reported (Mann-Whitney test).

	Time points	MCWD remaining mass (%)	A-MCWD remaining mass (%)
**PBS**	1 day	82.11 ± 1.47	100
	3 days	77.25 ± 5.09	100
	7 days	29.47 ± 7.93	100
	10 days	7.89 ± 7.93	99.32 ± 0.3
**Collagenase**	6 hours	28.85 ± 6.51	81.55 ± 3.39
	24 hours (1 day)	0	16.75 ± 7.01
	48 hours (2 days)	0	3.16 ± 1.03
	168 hours (7 days)	0	0

Table S3: MCWD and A-MCWD remaining mass (%) after degradation at different time points in physiological (PBS) and enzymatic (collagenase) conditions. All data are expressed as mean ± SD. Significant differences were evaluated with Mann-Whitney test (PBS: $p < 0.001$, collagenase: $p < 0.05$).
